# Adherence to Web-Based Self-Assessments in Long-Term Direct-to-Patient Research: Two-Year Study of Multiple Sclerosis Patients

**DOI:** 10.2196/jmir.6729

**Published:** 2017-07-21

**Authors:** Peter Joseph Jongen, Ingrid E.H Kremer, Elena Hristodorova, Silvia M.A.A Evers, Anton Kool, Esther M van Noort, Mickaël Hiligsmann

**Affiliations:** ^1^ Department of Community and Occupational Medicine University Medical Center Groningen University of Groningen Groningen Netherlands; ^2^ MS4 Research Institute Nijmegen Netherlands; ^3^ CAPHRI Care and Public Health Research Institute Department of Health Services Research Maastricht University Maastricht Netherlands; ^4^ Curavista BV Geertruidenberg Netherlands

**Keywords:** Internet, patients, multiple sclerosis, surveys and questionnaires, self-assessment, patient compliance, quality of life

## Abstract

**Background:**

Direct-to-patient research via Web-based questionnaires is increasingly being used. Missed data or delayed reporting of data may negatively affect the quality of study results. It is insufficiently known to what degree patients adhere to agreed self-assessment schedule over the long term and whether questionnaires are filled out in a timely manner.

**Objective:**

The objective of this study was to investigate patients’ adherence to a self-assessment schedule with low-frequency long questionnaires versus that with a high-frequency short questionnaire.

**Methods:**

In this study, the 36-item MS Impact Profile (MSIP) questionnaire measured (perceived) disabilities and the 54-item MS Quality of Life-54 (MSQoL-54) questionnaire measured health-related quality of life at 6-month intervals. Additionally, the 2-item Medication and Adherence (MA) questionnaire documented medication and adherence to disease-modifying medication every month. An experienced MS nurse assessed the Expanded Disability Status Scale (EDSS) score via phone. For both the self-assessment schedules, we calculated the percentage of patients who had completed all the questionnaires in the first 2 years (completion adherence), the percentage of patients who completed all the questionnaires within set time frames (interval adherence), the relationship between adherence and the EDSS score, and the timing of EDSS assessment.

**Results:**

Of the 331 patients who enrolled themselves, 301 patients completed at least one questionnaire. At month six (M6), M12, M18, and M24, the MSIP was completed by 83.4% (251/301), 71.8% (216/301), 68.1% (205/301), and 58.5% (176/301) of the patients, respectively; the MSQoL-54 by 82.1% (247/301), 71.8% (216/301), 66.8% (201/301), and 57.1% (172/301), respectively; and the MA questionnaire by 80.1% (241/301), 70.4% (212/301), 62.1% (187/301), and 53.5% (161/301), respectively. For the MSIP, 56.8% (171/301) of the patients were 2-year completion adherent; 55.5% (167/301) and 53.5% (161/301) of the patients were completion adherent for the MSQoL-54 and MA questionnaires, respectively. Whereas 85.5% (142/166) of the patients were interval adherent for the MSIP and MSQoL-54, 25.5% (41/161) were interval adherent for the MA questionnaire, with 73.9% (119/161) exceeding the maximum MA monthly interassessment interval. Completion adherence for the monthly short MA questionnaire was higher in patients with moderately high disability (EDSS 5.0-5.5) than for those with no or minimal disability (EDSS 0-2.5) (OR 5.47, 95% CI 1.08-27.69; P=.040). Completion adherence was also higher in patients with EDSS assessment within 6 months after baseline than in those with later assessment (OR 1.810, 95% CI 0.999-3.280; P=.050).

**Conclusions:**

The 2-year completion adherence to Web-based self-assessments did not differ between the low-frequency long questionnaires and a high-frequency short questionnaire, but the interval adherence was substantially higher for the low-frequency long questionnaires. Personal contact with a member of the research team regarding a clinically relevant professional-reported outcome early in the study might positively affect the long-term completion adherence in direct-to-patient studies.

## Introduction

Multiple sclerosis (MS) is a chronic inflammatory demyelinating and degenerative disease of the central nervous system, mainly affecting persons in young adulthood. In about 80% of the patients, the first phase is characterized by a pattern of recurrent episodes of symptoms (relapses), typically followed by complete or partial remissions. This phase of MS is referred to as relapsing remitting MS (RRMS) [1]. Although disease-modifying drugs (DMDs) reduce the frequency and severity of relapses, after about 20 years, most persons with RRMS progress to the secondary progressive (SP) phase, experiencing a steady and unstoppable increase in disability [2,3]. In about 10% to 15% of the patients, symptoms start insidiously and develop slowly without relapses. This form of MS is referred to as primary progressive MS (PPMS) [1]. In both SPMS and PPMS, the continuous increase in disability results mainly from degenerative processes. The multifocal localization of the lesions accounts for the wide variety of symptoms that may arise in the course of the disease; these symptoms often interfere with physical, cognitive, social, or occupational activities. Over the long term, the MS-related disabilities often represent a substantial burden to the patients and their environment.

To enable neurologists to better prognosticate the disease course in individual patients, they need to be informed in more detail about the degree of and variation in long-term disabilities. To obtain this information, patient-reported outcomes (PROs) are increasingly being used in addition to physician-based measures such as the Expanded Disability Status Scale (EDSS). A PRO is any report of the status of a patient’s health condition that comes directly from the patient, without interpretation of the patient’s response by a clinician or anyone else [4]. A PRO that is increasingly being used in clinical research is health-related quality of life (HRQoL), an overall measure of well-being from a patient’s perspective that provides a comprehensive measure of health status [5].

The Internet has empowered patients to directly participate in research projects without the involvement of their physician or physician’s setting [6,7]. The rapid adoption of the Internet by MS patients, the limited costs of Web-based contacts, and the easy access to large numbers of potential participants are all in favor of a direct-to-patient study design [6,8]. The data that can thus be obtained include PROs on various symptoms, (perceived) disabilities, HRQoL, and treatment, and these data may complement neurologist- or nurse-reported data about diagnosis, disease course, and magnetic resonance imaging [6].

In view of the scarcity of long-term data on (perceived) disabilities and HRQoL in MS patients in the Netherlands, we conceived the prospective, direct-to-patient, interactive, Dutch MS study [9]. The study participants enrolled themselves and agreed to complete two long questionnaires on (perceived) disabilities (36 items) and HRQoL (54 items) at 6-month intervals and a short questionnaire on medication and adherence to DMD treatment (two items) at monthly intervals [9].

One of the crucial aspects of long-term direct-to-patient research is the participants’ adherence to the predetermined assessment schedule. A reduction in the amount of data that patients provide may seriously affect the validity and meaningfulness of the study results [10,11]. Conceivably, the same amount of data can be acquired by the infrequent use of long questionnaires or the frequent use of short questionnaires. In fact, different factors might determine the adherence to a self-assessment schedule: a high-frequency short questionnaire could be bothersome to patients due to the frequent interference with their daily life or frequent confrontation with their disabilities and limitations, whereas a low-frequency long questionnaire might be cumbersome because of requiring more time to complete, and thus, potentially increasing MS-related fatigue.

In a previous patient-centered Web-based study involving RRMS patients who were included by their neurologists, we investigated the adherence to monthly Web-based self-assessments after the start of DMD treatment [12]. It was found that 75.5% of the patients completed two short questionnaires at all monthly time points over the course of 1 year, although only 1 in 5 patients adhered to the monthly intervals between consecutive self-assessments [12]. To gain information about the long-term adherence of MS patients to low-frequency completions of long questionnaires versus high-frequency completions of short questionnaires in a direct-to-patient research setting, this study analyzed the 2-year adherence data in the Dutch MS study [9]. Given the study design, participation in this study was not a priori integrated into patient care. As the embedding of research activities in care processes may positively affect patient adherence and adherence may decrease over time, it was hypothesized that at least for the high-frequency short questionnaire, the 2-year completion adherence would be less than 75%.

## Methods

### Dutch Multiple Sclerosis Study

The Dutch MS study is a prospective, Web-based, direct-to-patient, interactive study of long-term disabilities, perception of disabilities, and HRQoL in patients with MS in the Netherlands. The innovative study design is characterized by Web-based patient-driven enrollment, Web-based data acquisition, the use of PROs, and the use of personal study data by patients and authorized health care professionals for self-assessment and assessment, self-monitoring and monitoring, or multidisciplinary care. The objectives of the study, design, target population, recruitment, ethical aspects, data acquisition, technical aspects, outcome measures, assessment schedule, organization, and funding have been described in detail elsewhere [9].

Patients were informed about the study via websites of three patient organizations and of the MS4 Research Institute [13]. By regular mail, neurologists and MS nurses were sent an informative letter with patient brochures, which they were asked to hand out to their patients. The brochure was also sent to the patrons of the National MS Foundation, the Netherlands, as an attachment to the foundation’s quarterly journal and related mailings. In the journal, study information was presented by the principal investigator (PJ). Information about the study was published twice in health specials of large national and regional Dutch newspapers. The protocol was submitted to the Ethics Committee *Medisch Ethische Toetsing Onderzoek Patiënten en Proefpersonen* in Tilburg, the Netherlands (nr M379). The committee concluded that a review was not indicated, as the study did not qualify for being tested according to the Dutch Medical Research Involving Human Subjects Act [14,15]. The study is being conducted in agreement with the Declaration of Helsinki (Ethical Principles for Medical Research Involving Human Subjects version 2013; 64th World Medical Association General Assembly, Fortaleza, Brazil, October 2013) [16], and the Dutch Medical Research Involving Human Subjects Act [15].

Technically, the study is a modular application on the Curavista eHealth platform (Curavista bv, Geertruidenberg, the Netherlands), built on an Oracle database with Java-scripting, XML-applets, and AJAX protocols (Oracle Corporation, Redwood City, CA). Data processing is 256-bit encrypted with virtual private network tunneling. The databases and software are physically secured in a dedicated data center in the Netherlands [9]. On the day of the scheduled assessment, patients receive a notification by an email indicating that a questionnaire is available for completion. If the questionnaire is not completed on the scheduled date, reminders are sent after 4 and 7 days.

Disabilities, perceptions of disabilities, and HRQoL are measured every 6 months via the Multiple Sclerosis Impact Profile (MSIP) and the Multiple Sclerosis Quality of Life-54 (MSQoL-54) questionnaires. The MSIP is made available first and the MSQOL-54 one week later. The medication that is being taken and the adherence to DMD treatment are secondary outcomes; these are measured every month via the Medication and Adherence (MA) questionnaire. Every 6 months, the completion of the MA questionnaire coincides with the completion of the MSIP and MSQoL-54. The completion of the combined MSIP and MSQoL-54 takes about 30 to 45 min; completion of the MA questionnaire takes less than 5 min.

### Questionnaires

#### Multiple Sclerosis Impact Profile

The MSIP is a measure of MS-related disabilities and perception of disabilities with established psychometric properties [17,18]. It is based on the International Classification of Functioning, Disability and Health and reflects an objectified view of the prevalence and severity of the impact of MS. The MSIP comprises 36 questions assessing disability (Q1a-Q36a) and perception of disability (Q1b-Q36b) in the following domains: muscle and movement functions; excretion and reproductive functions; activities involving basic movements; activities of daily living; participation in life situations; environmental factors; mental functions; and the symptoms fatigue, pain, speech, and vision [17,18].

#### Multiple Sclerosis Quality of Life-54

HRQoL is assessed with the MSQoL-54 questionnaire, a psychometrically validated MS-specific multidimensional inventory of patient-centered health status [19]. The MSQoL-54 consists of the 36-item Short Form health survey as a generic core measure to enable comparisons with other patient populations and to the general population, supplemented with 18 additional questions exploring items relevant to MS patients in the areas of health distress (four items), sexual function (four items), satisfaction with sexual function (one item), overall quality of life (two items), cognitive function (four items), energy (one item), pain (one item), and social function (one item) [19].

#### Medication and Adherence Questionnaire

The MA questionnaire gives an update of medications that are taken, the number of DMD doses missed in the past month, and the date and reason of DMD discontinuation (if applicable).

### Disability Assessment by Phone

The EDSS is a widely used disability measure in MS. The EDSS quantifies disability in eight functional systems and allows neurologists and qualified nurses to assign a functional system score in each of these systems [20]. The functional systems are as follows: pyramidal, cerebellar, brainstem, sensory, bowel and bladder, visual, cerebral, and other. The EDSS steps 0.0 to 4.5 refer to MS patients who are fully ambulatory, and EDSS steps 5.0 to 9.5 are defined by the impairment to ambulation. A version of the EDSS that can be used as a structured interview by phone and has been validated for serial assessments in a research setting was used in the Dutch MS study [21,22]. All patients were sent an email in which they were asked if they agreed to a disability assessment via an interview by phone. The emails were sent in order of enrollment. If patients agreed, they were asked to provide information about the days of the week and the time of the day they were available for the assessment by phone. Patients were free regarding when to contact the study team and when to schedule the EDSS interview. In addition to answering the questions of the structured interview during the phone contact, the patients had the opportunity to ask study-related information or discuss study aspects with the assessing researcher, an experienced nurse specialized in MS.

### Study Outcomes

The outcomes of this analysis are the adherence to the assessment schedules of the MSIP, MSQoL-54, and MA questionnaires during the first 2 years of the study. Regarding the MSIP and the MSQoL-54, patients were classified as completion adherent for the respective questionnaire if they had performed all five scheduled 6-month assessments during the first 2 years. Patients were classified as completion adherent for the MA questionnaire if they had performed all scheduled monthly assessments during the first 2 years. Patients were classified as overall completion adherent if they had performed all scheduled MSIP, MSQoL-54, and MA assessments in this period.

Patients who were completion adherent for the MSIP or MSQoL-54 were classified as interval adherent if they met the following three criteria: (1) median interassessment interval was 180+10 days or less, (2) maximum interassessment interval was 180+20 days or less, and (3) month 24 (M24) completion was within 30 days after the scheduled date. Patients who were completion adherent for the MA questionnaire were classified as interval adherent if (1) the median interassessment interval was 30+3 days or less, (2) the maximum interassessment interval was 30+6 days or less, and (3) the M24 completion was within 30 days after the scheduled date.

### Statistical Analysis

The numbers of patients who completed the respective questionnaires at the various time points were calculated, as well as the intervals between two consecutive assessments and between the baseline and M24 assessment. The intervals between two consecutive assessments (days) were presented as mean, standard deviation (SD), median, minimum, maximum, and interquartile range (IQR). Friedman’s analysis of variance and the Wilcoxon signed-rank test were used to test whether the intervals for consecutive time points differed between the MSQoL-54, MSIP, and MA questionnaires. The numbers of patients who were completion- or interval adherent are expressed as the percentage of patients who actually started participating in the study by completing at least one of the questionnaires. To compare the completion adherence rates and interval adherence rates of the three questionnaires, Cochran’s Q test was performed. To test for significant associations between sex, age, EDSS score, and the timing of EDSS assessment on the one hand, and completion adherence and interval adherence regarding the low-frequency long questionnaires (MSIP, MSQoL-54) and the high-frequency short questionnaire (MA) on the other hand, we used logistic regression analysis. All tests were performed in Statistical Package for the Social Sciences for Windows version 24 (IBM Corporation, Armonk, NY).

The EDSS score was categorized into no to minimal disability (scores 0-2.5), fully ambulatory with moderate disabilities (scores 3.0-3.5), fully ambulatory with little to moderate effect on daily activities (scores 4.0-4.5), ability to walk about 100 to 200 m without aid and fully or severely impaired in performing daily activities (scores 5.0-5.5), ability to walk about 20 to 100 m with aid (scores 6.0-6.5), and severely disabled in walking or fully restricted to bed or chair (scores above 7.0). The timing of EDSS assessment by phone was dichotomized into assessment within 6 months after baseline self-assessment and later than 6 months after baseline self-assessment. A *P* value of .05 was applied for significance.

## Results

### Patients

A total of 331 patients had enrolled themselves in the study at least 2 years before the date of analysis (July 2015), from March 23, 2011 to March 15, 2012. Of these, 301 (90.94%) had actually started participating in the study by completing at least one questionnaire at baseline, whereas 30 (9.06%) patients had effectively not started participation. Of the 331 patients, 246 (74.32%) were female, 67 (20.24%) were male, and for 18 (5.40%) the sex was unknown. The mean age was 45.59 (SD 11.05) years, the median was 45.13, the minimum was 17.18, the maximum age was 70.57, and the IQR was 37.82-53.92 (N=310). Of the 301 patients who had completed at least one questionnaire at baseline, 234 (77.74%) were female and 67 (22.25%) were male. The mean age was 45.52 (SD 11.08), the median was 44.96, the minimum was 17.18, the maximum was 70.57, and the IQR was 37.82-53.92 (N=298).

### Completions

**Figure 1:**
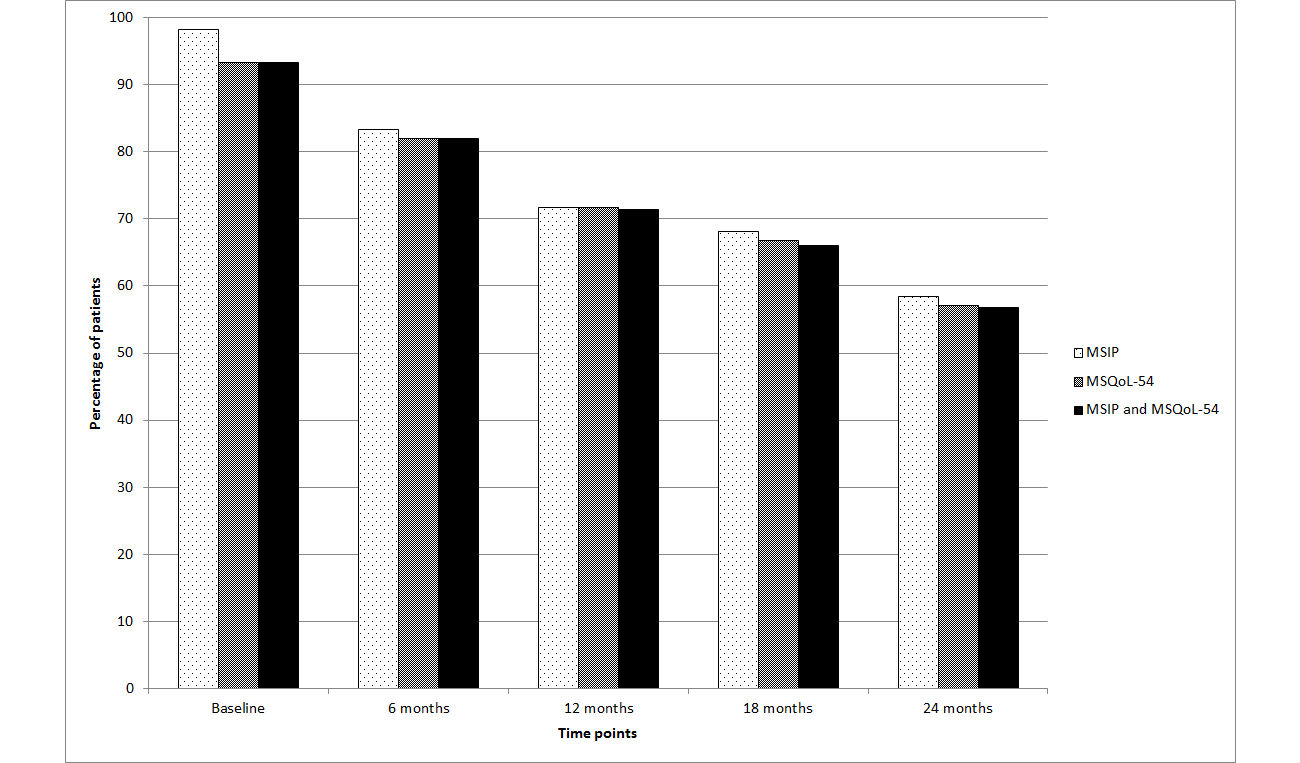
Percentages of patients who completed the Multiple Sclerosis Impact Profile and Multiple Sclerosis Quality of Life-54 questionnaires at baseline and at 6, 12, 18, and 24 months (N=301).

The numbers and percentages of patients who completed the MSIP at baseline, M6, M12, M18, and M24 were 296 (98.3%), 251 (83.4%), 216 (71.8%), 205 (68.1%), and 176 (58.5%), respectively, and the numbers and percentages of patients who completed the MSQoL-54 at baseline, M6, M12, M18, and M24, were 281 (93.4%), 247 (82.1%), 216 (71.8%), 201 (66.8%), and 172 (57.1%), respectively (Figure 1). The numbers and percentages of patients who completed both these questionnaires at baseline, M6, M12, M18, and M24 were 281 (93.4%), 247 (82.1%), 215 (71.4%), 199 (66.1%), and 171 (56.8%), respectively (Figure 1).

**Figure 2:**
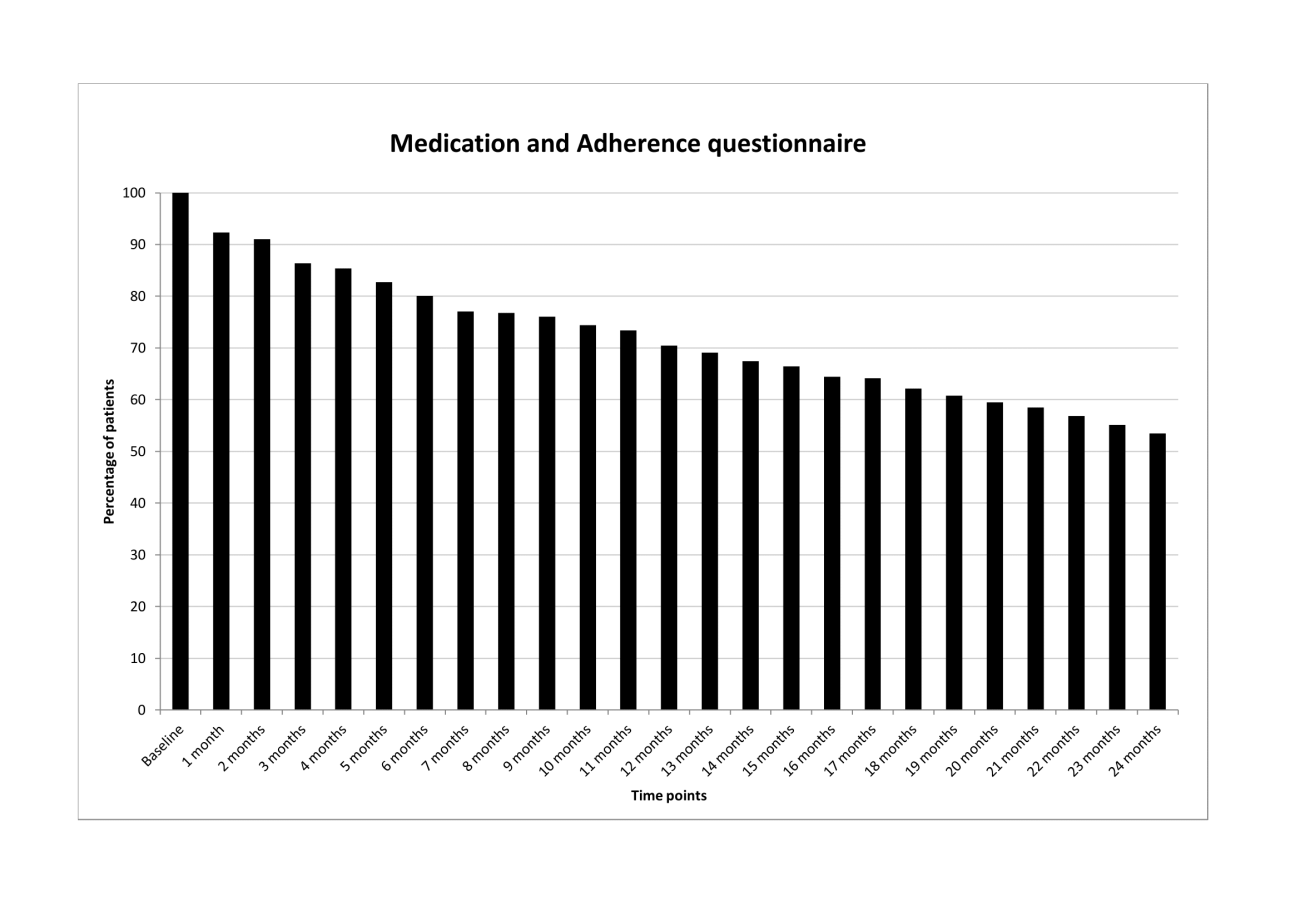
Percentages of patients who completed the Medication and Adherence questionnaire at baseline and at the various monthly time points (N=301).

Figure 2 shows the numbers of patients who had completed the MA questionnaire at various time points, expressed as the percentage of patients (N=301) who had started study participation. The numbers of patients who had completed the MA questionnaire at baseline, M6, M12, M18, and M24 were 301 (100%), 241 (80.1%), 212 (70.4%), 187 (62.1%), and 161 (53.5%), respectively.

Table 1 shows the numbers and percentages of patients who completed the MA questionnaire at all 25 time points, at 24 to one time point(s), or at no time point, irrespective of these being consecutive assessments.

The numbers and percentages of patients who completed the respective questionnaires at baseline and at M6, M12, M18, and M24 (five time points) at four, three, two, or one time point(s), or at no time point, irrespective of these being consecutive assessments, are shown in Table 2.

**Table 1:** Numbers and percentages of patients who completed the short Medication and Adherence questionnaire at all 25 time points, at 24 to one time point(s), or at no time point, irrespective of these being consecutive assessments (N=301).

Number of completions	n (%)
25 (all)	161 (53.5)
24	166 (55.1)
23	171 (56.8)
22	176 (58.5)
21	179 (59.5)
20	183 (60.8)
19	187 (62.1)
18	193 (64.1)
17	194 (64.5)
16	200 (66.4)
15	203 (67.4)
14	208 (69.1)
13	212 (70.4)
12	221 (73.4)
11	223 (74.1)
10	227 (75.4)
9	230 (76.4)
8	233 (77.4)
7	242 (80.4)
6	249 (82.7)
5	256 (85.0)
4	262 (87.0)
3	274 (91.0)
2	279 (92.7)
1	301 (100.0)
0	30

**Table 2:** Numbers and percentages of patients who completed the Multiple Sclerosis Impact Profile, Multiple Sclerosis Quality of Life-54, and Medication and Adherence questionnaires at baseline and at 6, 12, 18, and 24 months (five time points) at four, three, two, or one time point(s), or at no time point, irrespective of these being consecutive assessments (N=301).

Number of 6-month completions	Multiple Sclerosis Impact Profile	Multiple Sclerosis Quality of Life-54	Medication and Adherence
	n (%)	n (%)	n (%)
5 (all)	171 (56.8)	167 (55.5)	161 (53.5)
4	203 (67.4)	202 (67.1)	187 (62.1)
3	222 (73.8)	219 (72.8)	212 (70.4)
2	252 (83.7)	246 (81.7)	241 (80.1)
1	296 (98.3)	283 (94.0)	301 (100.0)
0	35	48	30

**Table 3:** Time intervals (days) between consecutive assessments.

Questionnaires		M0-M6		M6-M12		M12-M18		M18-M24		M0-M24	
Multiple Sclerosis Quality of Life-54												
	Mean (SD^b^)	177.82 (28.13)		188.42 (37.20)		180.25 (33.47)		180.75 (27.89)		724.05 (63.39)	
	Minimum	−42ª		99		−41ª		0		1	
	Maximum	266		545		369		257		825	
	Median (IQR^c^)	182 (180-184)		183 (182-185)		182 (181-184)		183 (181-186)		730 (729-733)	
Multiple Sclerosis Impact Profile												
	Mean (SD)	186.39 (35.86)		188.93 (34.69)		179.73 (31.44)		180.15 (28.27)		733.23 (21.13)	
	Minimum	114		166		−32ª		0		547	
	Maximum	738		552		374		305		852	
	Median (IQR)	183 (182-186)		183 (182.25-187)		182 (179-183.25)		183 (181-185)		731 (730-734)	
Medication and Adherence												
	Mean (SD)	188.10 (11.87)		192.26 (49.86)		220.67 (69.19)		186.46 (47.43)		763.75 (66.42)	
	Minimum	182		89		150		103		675	
	Maximum	285		550		700		370		1093	
	Median (IQR)	185 (182-188)		183 (180-188)		197 (189-216)		188 (152-200.5)		749 (718-776)	

^a^Negative value because some respondents (MSQoL-54, N=3; MSIP, N=1) did not complete the consecutive questionnaires chronologically.

^b^SD: standard deviation.

^c^IQR: interquartile range.

### Intervals

The intervals (days) between two consecutive assessments and between the baseline and M24 assessment (mean, SD, median, minimum, maximum and IQR values) are given in Table 3.

Median values for the intervals between two consecutive 6-month assessments ranged from 182 to183 days for the MSQoL-54 and the MSIP. The median time between baseline and M24 was 730 days for the MSQoL-54 and 731 days for the MSIP. For the MA questionnaire, the median values for interassessment intervals ranged from 30 to 32 days and the M24 assessment was at 749 days (median). The interval between baseline and M6 significantly differed between the three questionnaires (MSIP vs MSQoL-54: *z*=−5.37, *P*<.001; MSQoL-54 vs MA: *z*=−8.73, *P*<.001; MSIP vs MA: *z*=−8.05, *P*<.001), as did the M6-M12 interval between the MSIP and MSQoL-54 (*z*=−2.42, *P*=.014) and the M12-M18 intervals between MSQoL-54 and MA (*z*=−11.70, *P*<.001) and between MSIP and MA (*z*=−11.44, *P*<.001) (see Multimedia Appendix 1). For the M18-M24 interval, no significant differences were found between the three questionnaires. Significant differences in time from baseline to M24 were found between all three questionnaires (MSIP vs MSQoL-54: *z*=−4.04, *P*<.001; MSQoL-54 vs MA: *z*=−6.17, *P*<.001; MSIP vs MA: *z*=−5.59, *P*<.001).

### Adherence to Low-Frequency Long Questionnaires

Of the 301 patients who started with the study, 166 (55.1%) completed the MSIP and MSQoL-54 questionnaires at all five time points, and they were therefore completion adherent for the low-frequency long questionnaires. Of these, 159 (95.8%) completed the M24 questionnaires within 30 days of the scheduled date; 163 (98.2%) had a median interassessment interval of 180+10 days or less, and 143 (86.1%) had a maximum interassessment interval of 180+20 days or less. In all, 85.5% (N=142) of the patients who were completion adherent for the low-frequency long questionnaires were interval adherent for these questionnaires.

### Adherence to High-Frequency Short Questionnaire

Of the 301 patients who started participation, 161 (53.5%) completed the MA questionnaire at all monthly time points and were thus completion adherent for the high-frequency short questionnaire. Of these, 99 (62%) performed the M24 assessment within 30 days of the scheduled date; 153 (95.0%) had a median interassessment interval of 30+3 days or less; and 42 (26%) had a maximum interassessment interval of 30+6 days or less. In all, 26% (N=41) of the patients who were completion adherent for the high-frequency short questionnaire were interval adherent for this questionnaire.

### Overall Adherence

One hundred fifty-two (50.5%) patients were completion adherent for both the low-frequency long questionnaires and the high-frequency short questionnaire, and they were therefore considered overall completion adherent. In addition, 36 (24%) patients who were overall completion adherent were interval adherent for both the low-frequency long questionnaires and the high-frequency short questionnaire and were therefore considered overall interval adherent. In all, 12% of the patients who started with the study were overall completion and interval adherent.

### Comparative Analyses

The completion rates did not differ between the three questionnaires (Cochran’s Q test: X^2^=5.630; *P*=.063). From the above, it follows that 91.6% (152/166*100) of the patients who were completion adherent for the low-frequency long questionnaires were also completion adherent for the high-frequency short questionnaire. Conversely, 94.4% (152/161*100) of the patients who were completion adherent for the high-frequency short questionnaire were also completion adherent for the low-frequency long questionnaires. Moreover, 25% (36/142*100) of the patients who were interval adherent for the low-frequency long self-assessments were also completion adherent for the high-frequency short self-assessments. Conversely, 88% (36/41*100) of those who were interval adherent for the high-frequency short self-assessments were also completion adherent for the low-frequency long self-assessments.

There were no statistically significant differences between men and women regarding the completion and interval adherence to the low-frequency long questionnaires, the high-frequency short questionnaire, or regarding the overall adherence rates. Likewise, no association was found between age and adherence.

As for the EDSS, patients with an EDSS score of 5.0 or 5.5 were found to have higher odds of being completion adherent for the high-frequency short questionnaire than patients with an EDSS score of 0 to 2.5 (OR 5.47, 95% CI 1.08-27.69, *P*=.040). Moreover, patients who had an EDSS assessment within 6 months after baseline were more likely to be completion adherent for the high-frequency short questionnaire than those whose EDSS score was assessed later (OR 1.810, 95% CI 0.999-3.280, *P*=.050).

## Discussion

In recent years, the direct-to-subject approach is being applied increasingly in clinical studies, both in trials organized by clinical research organizations and in investigator-driven academic research [23,24]. This development is paralleled by a growing number of studies that make use of the Internet for the acquisition of patient-reported data. However, it is insufficiently known to what degree patients who enroll themselves in Web-based studies do indeed perform the scheduled assessments and whether they do so on time, especially over the long term. Such knowledge is relevant, as patients who prematurely discontinue their participation or those who provide data only infrequently or delayed may hamper the validity of the study results.

To obtain insight into patients’ long-term adherence to a self-assessment schedule in a setting of Web-based direct-to-patient research, we analyzed the numbers of completed questionnaires and the interassessment intervals in the first 2 years of the Dutch MS study regarding two low-frequency long questionnaires (MSIP, MSQoL-54) and one high-frequency short questionnaire (MA).

### Principal Findings

First, we found that about 56% of the patients completed the two long questionnaires at all five 6-month time points (MSIP: 56.8%, MSQoL-54: 55.5%); and second, that about 54% of the patients completed the short questionnaire at all 25 monthly time points. Third, we found that over 90% of the patients who completed all questionnaires for one type of assessment also completed all questionnaires for the other type; fourth, that the number of patients who completed the questionnaires decreased gradually over time, and, fifth, that the patients who completed all of the long questionnaires at 6-month intervals in a timely fashion by far outnumbered the patients who performed all of the monthly short self-assessments in time (85.5% vs 26%).

So, interestingly, over a 2-year period no difference was found in completion adherence (completion of all scheduled assessments) between the two less frequent long questionnaires and a more frequent short questionnaire. This was so despite the evident differences in patient burden: the short MA questionnaire had to be completed 5 times more frequently than the long MSIP and MSQoL-54, and the completion time of the latter was 6 to 9 times longer than that of the MA questionnaire. This suggests that completion adherence is influenced not so much by quantitative aspects like frequency of assessments and completion time but other factors. These factors could be the perceived relevance of the questionnaires’ content and the degree to which health care providers use the questionnaires’ outcomes in their disease management.

It may well be that patients’ adherence to the completion of Web-based questionnaires is influenced by the outcomes’ relevance for the disease management such as decisions on treatment initiation, continuation, or discontinuation. It is of note that one of the characteristics of the Dutch MS study is that patients may give health care professionals access to the completed questionnaires and the automatically generated scores. Although we suggested the study participants inform their neurologists, MS nurses, and other health care professionals about this option, only 21 patients authorized one or more health care professionals. Accordingly, we think that the low utilization of the interactive aspect of the study may also explain why at 2 years about 45% of the patients failed to complete all questionnaires.

Figure 2 suggests that the number of patients who completed the short monthly MA questionnaire at a given time point decreased on average by 2.4% per month, an exception being the decrease of 7.6% at the first interval. Remarkably, the decrease over time in the number of patients who completed the 6-month long questionnaires fits in with the pattern of decreasing completions of the MA questionnaire. There was another interesting observation: the less frequent long and more frequent short questionnaires showed almost identical completion percentages at M6, M12, M18, and M24. In combination with the fact that more than 90% of the patients who were completion adherent for one type of assessment were also completion adherent for the other type and the quasi-linear decrease in the number of completion adherent patients for both types of assessment, this observation suggests that nonadherent patients completed virtually all scheduled questionnaires up to a certain time point, at which they decided not to complete any more questionnaires. From patients’ phone calls to the help desk it became clear, rather unexpectedly, that participants were sometimes reluctant to complete the monthly MA questionnaire because no changes in medication occurred over longer periods or because they felt ‘spied on’ by the frequent assessments. It may therefore be hypothesized that a reluctance to complete one specific questionnaire may have affected not only the completion of that particular questionnaire but that of the other questionnaires as well.

Differences between the two types of assessment were found for interval adherence. Whereas the majority (85.5%) of patients were interval adherent for the low-frequency long questionnaires, only a minority (26%) was so for the high-frequency short questionnaire. The nonadherence in the latter group was mainly because of the fact that approximately 3 out of 4 patients (73.9%) exceeded the maximum interassessment interval of 30+6 days at least once and less so to 4 out of 10 (38.5%) patients performing the M24 assessment later than 30 days after the scheduled date. This difference in interval adherence may relate to the difference in assessment frequencies, as less frequent assessments lower the risk of one assessment exceeding the maximum interassessment interval. The difference may also be due to the predefined criteria for interval adherence: the allowed time window of 6 days for the monthly completions may have been too narrow for patients who, for example, because of an MS relapse or a concomitant disease, were temporarily unable to complete questionnaires. When comparing the intervals for the consecutive time points, no consistent differences were found between the MSQoL-54, MSIP, and MA questionnaires.

It was found that patients with the ability to walk about 100 to 200 m without aid and fully/severely impaired in performing daily activities (EDSS 5.0 or 5.5) were five times more completion adherent for the more frequent short questionnaire than patients with no or minimal disability (EDSS 0 to 2.5). We speculate that this may relate to the former patients being more housebound and thus possibly having more time at their disposal and the latter being more involved in familial, professional, and societal activities with less time for or interest in the regular completion of questionnaires. However, at higher EDSS scores (6.0 and higher), this association was not found, which could relate to the circumstance that cognitive and physical disabilities prevented these patients from performing moderately demanding tasks.

Interestingly, patients who—within 6 months after completion of the first questionnaire—had their disability assessed by an experienced MS nurse via phone were almost twice as likely to be completion adherent for the high-frequency short questionnaire than were those whose EDSS score was assessed later. This observation suggests that an early personal contact between the patient and a member of the research team—with the opportunity to ask questions about the study or about individual health status—may positively influence adherence to an assessment schedule.

### Comparison to Prior Work

Whereas a first experience has been reported with direct-to-patient recruitment for enrollment into clinical trials [25], to our knowledge no studies have investigated the adherence to Web-based assessments in long-term direct-to-patient research. In general, early discontinuation of study participation has been associated with various sociodemographic and health-related factors such as being male [26], black [27], having cognitive impairment [28,29], and experiencing difficulties in activities of daily living [28]. We did not find differences in completion or interval adherence between males and females. Our finding that patients with moderately high disability were more completion adherent for the frequent short questionnaire than were patients with no or minimal disability does not contradict a previous report on higher dropout rates in very ill persons. In MS patients, disability mostly results from impaired mobility and not from deficiencies in general health.

In this study, 64.0% of the patients had completed all monthly MA questionnaires 1 year after baseline. In a previous 1-year study in MS patients who started daily glatiramer acetate treatment, we found that 75.5% of the patients completed all monthly short questionnaires on fatigue (five items) and HRQoL (eight items) [12]. This higher percentage could relate to the content of the questionnaires: fatigue is a frequent and often debilitating symptom in MS that was expected to improve during glatiramer acetate treatment, whereas the documentation of medication and missed DMD doses may be less appealing to patients. Moreover, in the glatiramer acetate study, patients were included by their treating neurologists at the time of treatment initiation, whereas in this study, patients enrolled themselves at an arbitrary point in time. Nonetheless, the median monthly interassessment intervals (30-32 days) and the median baseline-M24 interval ([2x365]+19 days) in this study compare favorably with the median interassessment intervals (32-34 days) and the median baseline-M12 interval (365+52 days) in our previous study [12].

### Limitations

Our study has several limitations. First, we analyzed data from a study that was not primarily designed to (also) investigate the adherence to assessment schedules. Second, by comparing the adherence to low-frequency long questionnaires with adherence to a high-frequency short questionnaire, we investigated two variables simultaneously, and we therefore cannot identify the relative contribution of a questionnaire’s frequency and length to the adherence. Third, we confined ourselves to the analysis of formal aspects of the questionnaires and did not consider their content, so it may well be that irrespective of the assessment frequency, patients experienced questions about (perceived) disabilities (MSIP) as more disturbing and less motivating than questions about DMD adherence. Fourth, although the fairly even distribution of the Dutch MS study participants throughout the Netherlands suggests that the study group is representative of the Dutch MS population, this has not been demonstrated; moreover, relatively healthy information technology users and enthusiasts may be overrepresented in the study group. Fifth, in view of the direct-to-patient study design, we did not verify the MS diagnosis with the patients’ neurologists or whether the diagnosis was made according to the latest criteria.

As to the instruments we used, it is important to note that the e-versions of the questionnaires have not been validated. There is, however, a vast amount of literature showing that e-versions of questionnaires and scales are equivalent to paper-and-pencil versions and that both can be used interchangeably. This has been demonstrated, among others, for questionnaires about disability [30], symptoms [31,32], HRQoL [33,34], psychopathology [31,35,36], and psychology [37]. Against this background we thought it reasonable to apply e-versions of the MSIP, MSQoL-54, and the MA questionnaire. Moreover, should any discrepancies exist between paper and e-version of these questionnaires, these will be of minor relevance as we consequently used the e-versions throughout the study. As to the EDSS assessment, the scoring via interview by phone has been validated for serial assessments in research settings, but it is not interchangeable with the physician-derived EDSS, especially for the lower range of disability [22].

Finally, our definitions of completion and interval adherence were based on what we considered both realistic from a patient perspective and desirable from the researcher’s point of view. To be qualified as completion adherent, we required patients to have completed all the questionnaires. Yet, the completion of five long questionnaires may be easier for patients to realize than the completion of 25 short questionnaires over the same time period. Additionally, from a research perspective, it may be questioned whether the missing of 1 out 5 or even 1 out of 25 assessments substantially hampers the data quality. Moreover, the time windows for interval adherence used by us are debatable and, in general, criteria for interval adherence will depend on the phenomenon under study and the time span covered by a questionnaire.

### Conclusions

In analyzing the 2-year adherence to self-assessments in the direct-to-patient Dutch MS study, we found no differences in completion adherence (completion of all scheduled questionnaires) between the two low-frequency long questionnaires versus the high-frequency short questionnaire; however, the interval adherence (completion of questionnaires within predefined time frames) was considerably higher for the low-frequency long questionnaires. Moreover, patients with moderately high disability were more likely to be completion adherent for the high-frequency short questionnaire than patients with no or minimal disability, as were the patients who within 6 months after completion of the first questionnaire had their disability assessed by an experienced MS nurse via phone in comparison with those who had their assessment later. The latter observation may suggest that in Web-based direct-to-patient research, personal contact with a member of the research team or feedback on a clinically relevant, professionally reported outcome early in the study may positively affect the long-term adherence to self-assessments.

## Appendix

Multimedia Appendix 1 Comparison of time intervals between the MSQoL-54, MSIP, and MA questionnaires.

## Abbreviations

DMD: disease-modifying drug
EDSS: Expanded Disability Status Scale
HRQoL: health-related quality of life
IQR: interquartile range
MA: medication and adherence
MS: multiple sclerosis
MSIP: Multiple Sclerosis Impact Profile
MSQoL-54: Multiple Sclerosis Quality of Life-54
PPMS: primary progressive multiple sclerosis
PRO: patient-reported outcome
RRMS: relapsing-remitting multiple sclerosis
SPMS: secondary progressive multiple sclerosis
SD: standard deviation
