# Supplementary material for: Adherence to Web-Based Self-Assessments in Long-Term Direct-to-Patient Research: Two-Year Study of Multiple Sclerosis Patients
Source: J Med Internet Res. 2017 Jul 21;19(7):e249. doi: 10.2196/jmir.6729 (PMC5544895; doi:10.2196/jmir.6729)
Supplement: Multimedia Appendix 1 [file jmir_v19i7e249_app1.pdf]

Comparison of time intervals between the MSQoL-54, MSIP, and MA questionnaires

Comparison of time intervals	z[a]	*P*	z[a]	*P*	z[a]	*P*	z[a]	*P*	z[a]	*P*
MSQoL-54 vs MSIP	-5.37	<.001	-2.42	.014	-1.44	0.150	-0.82	.412	-4.04	<.001
MSQoL-54 vs MA	-8.73	<.001	-0.53	.593	-11.70	<.001	-0.03	.975	-6.17	<.001
MSIP vs MA	-8.05	<.001	-1.32	.182	-11.44	<.001	-0.01	.933	-5.59	<.001

[a]z: standardized Wilcoxon signed-rank value
